# Postnatal care services in rural Zambia: a qualitative exploration of user, provider, and community perspectives on quality of care

**DOI:** 10.1186/s12884-023-05350-w

**Published:** 2023-01-18

**Authors:** Cephas Sialubanje, Jeanette L. Kaiser, Thandiwe Ngoma, Lawrence Mwananyanda, Rachel M. Fong, Davidson H. Hamer, Nancy A. Scott

**Affiliations:** 1 School of Public Health, Levy Mwanawasa Medical University, Lusaka, Zambia; 2 Department of Global Health, Boston University School of Public Health, Boston, MA USA; 3 Department of Research, Right to Care Zambia, Lusaka, Zambia; 4 Section of Infectious Diseases, Department of Medicine, Boston University School of Medicine, Boston, MA 02118 USA

**Keywords:** Postnatal care, Quality, Zambia

## Abstract

**Background:**

Postnatal care (PNC) is an important set of services offered to the mother and her newborn baby immediately after birth for the first six weeks to prevent maternal and neonatal complications and death. This qualitative study explored user and provider perspectives on quality of PNC services in the selected health facilities within the context of the Maternity Homes Access in Zambia project in the Saving Mothers Giving Life districts in rural Zambia.

**Methods:**

Between October 2018 and February 2019, forty focus group discussions (FGDs) (*n* = 160 participants) and twelve in-depth interviews (IDIs) were conducted in four districts in Southern and Eastern provinces. FGDs comprised women who delivered within the last year, fathers, community elders, and volunteers. IDIs comprised health workers at facility, district, and provincial levels. Data were analysed using content analysis guided by the international quality of care domains derived from the World Health Organization quality of care framework. Findings were triangulated to understand perceptions.

**Results:**

Overall, study participants perceived PNC services to be beneficial. Nevertheless, respondents had mixed feelings on the quality of PNC services and expressed a stark difference in their perception of factors affecting service quality. Service users described challenges arising from ineffective communication about the new PNC guidelines, and non-adherence of service providers to quality standards regarding respect, preservation of dignity and emotional support. Other factors were long waiting hours, small examination rooms providing inadequate privacy, and low levels of confidentiality. In contrast, service providers attributed poor service quality to various health system-related factors including low staffing levels, dysfunctional referral services, low supply of essential medicines, supplies, vaccines and equipment for optimal routine emergency obstetric and newborn care and management of complications.

**Conclusion:**

These findings highlight important intervention opportunities to improve quality of PNC services in Zambia through better communication and raising awareness on PNC guidelines, respect, preservation of dignity and emotional support to mothers. Interventions should also focus on addressing contextual health system challenges including staffing levels, supply chain for essential medicines and commodities, shortening waiting time, and ensuring functional referral system.

**Supplementary Information:**

The online version contains supplementary material available at 10.1186/s12884-023-05350-w.

## Background

Despite the continued drop in global maternal mortality since 1990 [1], low- and middle-income countries (LMICs) still experience high maternal mortality ratios (MMR), accounting for 94% of maternal deaths worldwide [2]. These deaths are particularly concentrated in sub-Saharan Africa and South Asia, which account for 86% of the global burden of maternal deaths [1]. Zambia is an African country with high MMR (252 deaths per 100 000 live births) and neonatal mortality (23.5 deaths per 1000 live births) [3]. Globally, maternal deaths account for 10% of all deaths among women of reproductive age (15–49 years) [1, 2]. Most maternal deaths occur during childbirth (11–17%) and the first 48 h postpartum (50–71%) and are due to complications of pregnancy, delivery and unsafe abortions − including haemorrhage, hypertensive disorders, sepsis, and obstructive labour [1, 2]. Nevertheless, most maternal deaths are preventable when women have access to quality and timely postnatal care (PNC) [4–6].

The World Health Organisation (WHO) defines PNC as the care provided to the mother and her new-born child immediately after birth of the placenta and for the first six weeks (42 days) postpartum [5]. Prompt and quality PNC is a critical phase in the lives of mothers and newborns [6] for both the prevention and treatment of complications arising from pregnancy and delivery. PNC has the potential to avert a substantial proportion of maternal and perinatal mortality and morbidity. It also provides the mother with important information on family planning, well-baby care, umbilical cord care, HIV and malaria prevention, and infant nutrition [5]. To improve maternal and newborn health outcomes, essential and life-saving PNC interventions should be accessible to all individuals and of high quality, meeting the key domains of the WHO quality of care framework [7], which include evidence-based practices, actionable information systems, effective communication, functional referral systems, respect and preservation of dignity, emotional support, competent and motivated human resources, and availability of essential physical resources [5–7]. In addition, the services must be safe, effective, timely, efficient, equitable, and people-centred [5–7]. Access to quality PNC has remained low in most LMICs including Zambia, particularly in rural areas [8–11]. The latest Zambia demographic Zambia (ZDHS) [3] shows that one-fifth (21%) of rural women deliver at home and only 64% of these receive PNC within the first hour after delivery; more than 40% do not receive any PNC within the first 48 h postpartum. Moreover, for the women that do receive early PNC, available evidence suggests that the services provided in health facilities are low quality [12–15]. Studies conducted in LMICs show that, although women do receive PNC services from skilled providers in health facilities, these services are untimely, inconsistent, often inadequate and of low quality [12–15] and most women who go for PNC do not receive many the required services [16–18]. Some of the reported reasons for the poor PNC service quality include staff shortages, unskilled health workers, a lack of in-service training, and supervision and mentorship [19].

We conducted a qualitative study to explore user, provider, and community perspectives on the quality of PNC services provided in rural districts of Southern and Eastern Provinces, Zambia. The information on service quality provided here will help fill the knowledge gap on the quality of PNC services in rural Zambia and help guide Ministry of Health (MoH) policy on and provision of PNC services in the country.

### Theoretical framework

To study user, provider, and community perspectives on the quality of PNC services, we used the WHO framework for improving the quality of care for mother and newborn around the time of childbirth. The framework contains eight standards or domains of quality of care that should be assessed, improved, and monitored within the health system. Each standard of care has two main components: the quality statement and the quality measure. A quality statement sets out the requirements to achieve compliance with the standard; quality measures provide objective evidence for determining whether or not the requirements have been met. The eight domains of quality of care and their definitions are shown in the Table 1 below:

**Table 1:** WHO framework for improving the quality of care for mother and new-born around the time of childbirth

WHO quality of care standard	Definition
Evidence-based practices for routine care and management of complications	Every woman and newborn receives routine, evidence-based care and management of complications during labour, childbirth and the early postnatal period, according to WHO guidelines
Actionable information systems	The health information system enables use of data to ensure early, appropriate action to improve the care of every woman and newborn
Functioning referral systems	Every woman and newborn with condition(s) that cannot be dealt with effectively with the available resources is appropriately referred
Effective communication	Communication with women and their families is effective and responds to their needs and preferences
Respect and preservation of dignity	Women and newborns receive care with respect and can maintain their dignity
Emotional support	Every woman and her family are provided with emotional support that is sensitive to their needs and strengthens the woman’s capabilit
Competent, motivated personnel	For every woman and newborn, competent, motivated staff are consistently available to provide routine care and manage complications
Availability of essential physical resources	The health facility has an appropriate physical environment, with adequate water, sanitation and energy supplies, medicines, supplies and equipment for routine maternal and newborn care and management of complications

## Methods

### Study design

This was a cross-sectional, qualitative study employing focus group discussions (FGDs) with PNC service users and community-level stakeholders, and in-depth interviews (IDIs) with PNC service providers. FGDs were conducted in April and May, 2018; IDIs were conducted in February, 2019. FGDs have been used in public health research for over three decades [20]. They aim to explore participants’ experiences, beliefs and attitudes towards a target behaviour, by using group processes to stimulate responses and gain insights through participants’ exchanging views, questioning and challenging one another [21]. IDIs enable the researcher to understand participants' lived experiences through their own words and perspectives [22, 23]. Use of both FGDs and IDIs allows for in-depth exploration and understanding of various aspects regarding the subject under investigation. The approach also allows for triangulation and corroboration of the FGD and IDI findings, which, in turn, increases the internal validity of the study [24–26].

### Study setting

The study was conducted in four rural districts of Southern (Choma, Kalomo, and Pemba) and Eastern (Nyimba) Provinces at ten primary health centres within the Maternity Homes Access in Zambia (MAHMAZ) project. The MAHMAZ project was a quasi-experimental controlled before-and-after intervention design trial [27], implemented by Boston University and Right to Care Zambia (2015 to 2018) to evaluate the impact of newly constructed maternity waiting homes (MWHs) on reducing the distance barrier and increasing access to quality maternal health care services for rural women in Zambia [28, 29]. The MAHMAZ project did not directly address PNC services offered in the health facility but did construct new MWHs or upgrade existing ones; they also offered beds for postnatal women if they wanted to remain close to the health facility for PNC. MAHMAZ operated in the Saving Mothers Giving Life (SMGL) project districts [30] − a five-year public–private partnership implemented in the selected districts of Zambia and Uganda between (2012 to 2016) as part of a concerted response by the U.S. government through President Barack Obama’s Global Health Initiative. SMGL’s goal was to reduce maternal deaths by up to 50% in targeted districts in Uganda and Zambia—particularly during the critical window during labour, delivery, and the first 24–48 h postpartum when an estimated 2 of every 3 maternal deaths and 45% of neonatal deaths occur [30]. To achieve this goal, SMGL employed a systems approach focused on the health district level to ensure that every pregnant woman had access to clean and safe normal delivery services and, in the event of an obstetric complication, lifesaving emergency care within 2 h. The model served to strengthen the existing public and private health networks within each district and integrated maternal and newborn health services with HIV services. It provided intensive supply and demand side interventions focusing on improved staffing levels, medical stock availability and demand generating activities implemented by Safe Motherhood Action Groups (SMAGs) [31]. SMAGs are community-based volunteer groups (traditional birth attendants, community health workers, and local traditional and religious leaders) that aimed to reduce critical delays that occur at household level with regard to decision-making about seeking life-saving maternal healthcare at health facilities [31]. They were selected and trained by SMGL to encourage women to seek care during pregnancy and the critical first few days of life postpartum. SMAGs taught pregnant women and their spouses about the importance of having a birth plan, delivering in a health facility, and practicing healthy behaviours during pregnancy and early childhood. They also offered guidance and instructions to pregnant women regarding use of MWHs, decision-making process regarding childbirth preparedness and place of delivery and the importance of attending PNC visits. SMGL phase 1 (2012–2013) project results showed a 31% increase in access to and coverage of emergency obstetric and newborn care (EmONC), a 44% increase in the number of women delivering in health facilities, a 41% reductions in mothers dying across target districts, a 38% decline in health facility maternal mortality, and a 36% decrease in total stillbirths in the facility along with a decrease in perinatal mortality [32].

According to the most recent census conducted in 2010, the populations of the study districts were approximately 250,000 each for Kalomo and Choma districts (which administratively included Pemba district at the time) and approximately 77,000 for Nyimba district [33, 34]. The districts are all primarily rural, ranging from 76% (Choma/Pemba) to 93% (Kalomo) of the population living in rural areas [33, 34]. The health system of the districts comprises hospitals, health centres and several health posts. The primary stakeholders in the maternal health programmes are the Ministry of Health, Churches Health Association pf Zambia (CHAZ), non-governmental organisations, community leaders, and various community-based volunteers in health, including traditional birth attendants and SMAGs [35].

Zambia implemented new PNC guidelines in 2014 (see Table 2) [36], which sought to address the timing, number, and place of postnatal contacts, and content of PNC for all mothers and babies during the six weeks postpartum [37–39]. After an uncomplicated vaginal delivery in a health facility by a skilled birth attendant, which is strongly recommended by the WHO, women and their new-borns are advised to remain within the health facility for a minimum of 24 h for observation of danger signs, and prevention and treatment of postpartum complications, such as excessive bleeding, raised blood pressure or eclampsia. Women who give birth at home are encouraged to visit the health facility within 24 h postpartum. Subsequent visits are at day 2–3, day 7–14, and day 42 postpartum [37–39]. This changed previous guidelines which required women to remain in the hospital for a shorter period of 6 h postpartum and return to the health facility after 6 days and 6 weeks.

**Table 2:** 2013 WHO Recommendations on Postnatal care

**1. Discharge and timing of postnatal contacts**
** 1.1. Timing of discharge from a health facility after birth** • After an uncomplicated vaginal birth in a health facility, healthy mothers and newborns should receive care in the facility for at least 24 h after birth
** 1.2. Number and timing of postnatal contacts** • If birth is in a health facility, mothers and newborns should receive postnatal care in the facility for at least 24 h after birth • If birth is at home, the first postnatal contact should be as early as possible within 24 h of birth • At least three additional postnatal contacts are recommended for all mothers and newborns on day • Day 3 (48–72 h) • Between days 7–14 after birth • 6 weeks after birth
** 1.3. Home visits for postnatal care** • First week after birth for care of the mother and newborn
**2. PNC for the new-born**
** 2.1. Assessment for the baby** • Feeding • History of convulsions • Breathing, severe chest in-drawing • Spontaneous movement • Fever (temperature ≥ 37.5 °C), low body temperature (temperature < 35.5 °C) • Jaundice in first 24 h of life, or yellow palms and soles at any age
** 2.2. Exclusive breast feeding** • All babies should be exclusively breastfed from birth until 6 months of age • Mothers should be counselled and provided support for exclusive breastfeeding at each postnatal contact
** 2.3. Cord care**: High neonatal mortality settings (30 or more neonatal deaths per 1000 live births) • 4% chlorhexidine at delivery • Daily chlorhexidine (7.1% chlorhexidine digluconate aqueous solution or gel) application to the umbilical cord stump during the first week of life recommended for newborns who are born at home in settings
** 2.4. Cord care**: newborns in health facilities and at home in low neonatal mortality settings • Clean, dry cord care for • Use of chlorhexidine in these situations may be considered only to replace application of a harmful traditional substance, such as cow dung, to the cord stump
** 2.5. Other PNC services care for the newborn** • Bathing should be delayed until 24 h after birth. If this is not possible due to cultural reasons, bathing should be delayed for at least six hours • Appropriate clothing of the baby for ambient temperature is recommended • This means one to two layers of clothes more than adults, and use of hats/caps • The mother and baby should not be separated and should stay in the same room 24 h a day • Communication and play with the newborn should be encouraged • Immunization should be promoted as per existing WHO guidelines • Preterm and low-birth-weight babies should be identified immediately after birth and should be provided special care as per existing WHO guidelines
**3. Assessment of the mother**
** 3.1. First 24 h after birth** • All postpartum women should have routine and regular assessment of the following during the first 24 h starting from the first hour after birth: • Vaginal bleeding • Uterine contraction • Fundal height • Temperature • Heart rate (pulse) • Blood pressure should be measured shortly after birth. If normal, the second blood pressure measurement should be taken within six hours • Urine void should be documented within six hours
** 3.2. Beyond 24 h after birth** • Enquiries about general well-being and assessments regarding: • Micturition and urinary incontinence • Bowel function • Healing of any perineal wound • Headache • Fatigue • Back pain • Perineal pain and perineal hygiene • Breast pain • Uterine tenderness • Lochia • Breastfeeding progress • Emotional well-being • Family and social support they have and their usual coping strategies for dealing with day-to-day matters • All women and their families/partners should be encouraged to tell their health care professional about any changes in mood, emotional state and behaviour that are outside of the woman's normal pattern
**4. Counselling**
** 4.1. All women should be counselled on:** • Nutrition • Hygiene, especially handwashing • Birth spacing and family planning. Contraceptive options should be discussed, and contraceptive methods should be provided if requested • Safer sex including use of condoms
4.2. In malaria endemic areas, mothers and babies should sleep under insecticide-impregnated bed nets
4.3. All women should be encouraged to mobilize as soon as appropriate following the birth. They should be encouraged to take gentle exercise and make time to rest during the postnatal period
**5. Iron and folic acid supplementation** • Iron and folic acid supplementation should be provided for at least three months
**6. Prophylactic antibiotics** • The use of antibiotics among women with a vaginal delivery and a third or fourth degree perineal tear is recommended for prevention of wound complications • There is insufficient evidence to recommend the routine use of antibiotics in all low-risk women with a vaginal delivery for prevention of endometritis
**7. Psychosocial support** • Psychosocial support by a trained person is recommended for the prevention of postpartum depression among women at high risk of developing this condition

### Sampling strategy

Ten of the MAHMAZ sites were purposively selected for inclusion in the FGD and IDIs. Selection of the sites for inclusion was based on the health facility’s capacity to provide basic emergency obstetric and newborn care (BEmONC) including PNC services. FGD respondents from villages of varying distances to the health facility were purposively selected with assistance from SMAG members. Villages were stratified into two groups: those which were located within 10 km and those which were more than 10 km from the health facility. Stratification on distance was done in order to select a balanced number of participants regarding access to PNC services provided in health facilities. This was in line with the MAHMAZ project which focused on provision of MWHs to increase access to maternal health care services provided in health facilities. To be included in the FGDs, participants needed to be: a) pregnant at any gestation or recently delivered women (within 1 year; b) men with child under one year; c) SMAG members or TBAs; or d) elders (> 54 years) or mothers-in-law. IDI participants were purposively selected from the two provincial health offices, the four district health offices, and the ten health facilities. To be included, IDI participants needed to be: a) a maternal and child health (MCH) coordinator at the provincial and/or district level, or b) PNC providers from the selected health facilities. Selection of IDI participants was based on the individual’s position at the health facility, district, or provincial health office, experience in MCH policies and activities in the relevant sites, and availability during the times of data collection.

### Study population

FGD participants were included based on the following criteria: age 18 years or older; women who were pregnant (any gestation age) or had recently delivered (gave birth in the 12 months prior to the FGD); men with a child aged less than 12 months; SMAG member; community elder (aged 54 years or more). To participate in the IDIs, respondents needed to be health staff providing PNC services in a health facility or health staff in charge of maternal and child health (MCH) services at the district or provincial health office levels. Individuals aged less than 15 years and those who resided less than three months in the area were not eligible for participation in the FGDs or IDIs. Individuals who were unable or unwilling to provide informed consent were excluded from the study, as well as minors (15–17 years of age) without a guardian able or willing to provide assent.

### Instrument design

FGD and IDI data were collected using two separate paper-based interview guides which were prepared by the research team and included the following domains: awareness, utilization, and quality of MWHs, barriers and facilitators to facility delivery, preparedness and costs for delivery, quality of ANC services, and quality of PNC services. Only the PNC quality domain was analysed for this study. Questions for this domain were structured using the WHO quality of care framework (Table 1). Each question included probes to increase the depth of participant responses. To make it easy for the respondents, the domains were combined into four themes (Table 1). One domain, an actionable information system, was not assessed. Each instrument included a demographics section. Both the FGD and IDI interview guides were piloted during training of the research assistants and revised accordingly before starting the actual data collection exercise.

### Data collection procedures

A total of 20 FGDs were conducted with each of the four respondent groups: 10 with pregnant or recently delivered women; 5 with men with a child aged less than 12 months; 2 with SMAG members; 3 with community elders) at each of the ten study sites. FGDs were conducted by a pair of research assistants trained in interview techniques, the interview guide, and in ethical conduct of research involving human subjects. FGDs were conducted in the local languages of Tonga and Nyanja and were audio recorded. Each FGD was conducted at the health facility, included 6 to 8 participants, and lasted between 2.5 and 3 h. The PNC component lasted between 45 and 60 min. In addition, a total of twelve IDIs were conducted with six midwives from BEmONC health facilities, four district health office managers, and two provincial health office managers (Table 3). IDIs with midwives were conducted by research assistants; those with district and provincial managers were conducted by one of the research team members. All IDIs were conducted in English and audio recorded.

**Table 3:** Focus Group Discussions (*n* = 300) and IDIs

**FGDs**	**Number**	**IDIs**	**Number**
Pregnant or Recently Delivered Women	10	District MCH coordinators	4
Men with child under one year	5	Provincial MCH coordinators	2
SMAGs and TBAs	3	Facility PNC providers	6
Elders and Mothers-in-law	2		
Total	20		12

### Data management and analysis

Audio recordings of the FGDs and IDIs were transcribed and translated into English. A codebook was developed using a deductive approach [40] in which sub-themes were derived from the instrument questions and WHO quality of care framework. Similar statements were coded to the same nodes and nodes were grouped into themes. To allow for comparison of responses among FGD respondent type and between service users and providers, sets were created which allowed for comparison of the data by respondent attributes. Coding and analysis were conducted in Nvivo 11 MAC by a research team member (CS), who conducted four IDIs with MCH coordinators but was not part of the MAHMAZ project implementation team (CS) and was not involved in the FGD data collection, transcription, or translation processes. CS was attached to the MAHMAZ project for his research as a postdoctoral Fogarty fellow under a consortium of Harvard University, Boston University, Northwestern University, and University of New Mexico. Due to logistical and administrative challenges it was not possible to have multiple coders from other research team members. However, the codebook was shared with other research team members to ensure credibility of the coding process and validity of findings. Descriptive statistics and frequencies were used to summarise the respondent demographic data using SPSS Statistics 21. The most frequently discussed qualitative themes are presented by respondent type, supported by illustrative quotations.

## Results

### Respondent demographics

A total of 172 respondents participated including 160 (93.0%) in the FGDs and 12 (7.0%) in the IDIs. More than 3 in five (64.5%) of the respondents were female, and the majority (82.6%) were married. On average, pregnant women were younger (mean age: 25.7 years, SD 5.7) than men (mean age: 30.8, SD 8.2), SMAG members (mean age: 48.1, SD 9.8) and community elders (mean age 63.3. SD 8.5). The mean age of PNC providers and health systems staff was 38.1 (SD 9.2) years. The mean age of PNC providers and health systems staff was 38.1 (SD 9.2) years. More than half (58.8%) of the women FGD respondents were pregnant and 41.3% were recently delivered. Of the SMAG members, 6 (37.5%) were community health workers and 10 (62.5%) were SMAGs volunteers (Table 4).

**Table 4:** Demographic characteristics

**Characteristic**	**Community Members**					**Health System Staff** *N* = 12
	Women *N* = 80	Men *N* = 40	SMAGs *N* = 16	Elders *N* = 24	Total *N* = 160	
Female, n (%)	80 (100)	0 (0.0)	9 (56.2)	13 (54.2)	102 (63.8)	9 (75.0)
Age, mean (SD)	25.7 (5.7)	30.8 (8.2)	48.1 (9.8)	63.3 (8.5)	34.9 (15.4)	38.1 (9.2)
Marital status, n (%)						
* Married/cohabiting*	65 (81.3)	37 (92.5)	13 (81.3)	17 (70.8)	132 (82.5)	10 (83.4)
* Divorced/separated/widowed*	4 (5.0)	0 (0.0)	3 (18.8)	7 (29.2)	14 (8.8)	1 (8.3)
* Never married*	11 (13.8)	3 (7.5)	0 (0.0)	0 (0.0)	14 (8.8)	1 (8.3)
Delivery location (self or spouse), n (%)						
* Health facility/hospital*	78 (97.5)	35 (87.5)	-	-	113 (94.2)	-
* Home/other*	2 (2.5)	5 (12.5)	-	-	7 (5.8)	-
* Gravid status*						
* Currently pregnant*	47 (58.8)	-	-	-		
* Recently delivered*	33 (41.3)	-	-	-		
* SMAG members*						
* SMAG*	-	-	10 (62.5)	-	10 (62.5)	
* CHW*	-	-	6 (37.5)	-	6 (37.5)	

### Effective communication on PNC services

Community members and health systems staff (provincial and district managers and health facility staff) were unanimous on the benefits of PNC visits. During PNC visits, women and their newborn babies receive important information about child nutrition, breastfeeding and care, childhood vaccines and immunization schedule, growth monitoring and promotion, under five clinic and family planning. They also receive information about disease prevention such as HIV, tetanus, malaria, and others. Nevertheless, most mothers, men, and community elders were dissatisfied with the way health workers communicated the new guidance for PNC visit timings. They expressed ignorance on the timing of visits specified in the new guidelines. Further, participants argued that, during health promotion classes, most nurses/midwives referred to the old PNC schedule. Similarly, some IDI participants from health facilities reported not receiving any training on the new PNC schedule and that district health staff did not disseminate the new guidelines; others mentioned that their colleagues did not share the information when they returned from district orientation meetings. In contrast, some health systems staff and some mothers were aware of the change in the PNC timing of visits in the new guidelines. They argued that they had shared the guidelines to the health facilities and that trainings on the new guidelines were conducted.

“*Most of us were not trained in the new PNC guidelines. Our colleague who went for training did not orient us and [that person] has now retired. This makes it difficult for us to teach the mothers [on the new guidelines] when they come for PNC*”—Midwife, IDI participant, intervention site, Kalomo district

Consequently, the lack of awareness on the new guidelines made most mothers not follow the PNC schedule. For example, despite being unanimous on the need for a mother and her baby to remain in the PNC ward for observation after delivery, participants did not agree on the duration. Those who were aware of the new guidelines explained that mothers needed to remain in the postnatal ward for a minimum of 24 h. By contrast, most mothers and community elders who were unaware of the new guidelines argued that the woman should only stay for 6 h postpartum and that only women with complications should stay longer or be referred to the hospital for specialized care.

“*After delivery, we wait for 6 hours before we are allowed to go home” -* Mother, FGD participant, intervention site, Choma district

Similarly, most community members did not know when women were supposed to return for their second and third PNC visits; they were only aware of the old PNC schedule about the 6–6-6 rule − remaining in the postnatal ward for 6 h, and returning after 6 days and 6 weeks postpartum. Only a few mothers and community elders knew the new guidelines that women were expected to return within 48–72 h and after 7–14 days. Moreover, very few reported returning for the second and third visits according to the PNC schedule.

“*Most mothers don’t follow the new guidelines; they still follow the old ones and return at 6 days and 6 weeks”* - Midwife, IDI participant, control site, Pemba district

In contrast, health managers from the two provincial health offices confirmed receiving the new guidelines from the MOH and that they had distributed them to the district health offices (DHO). DHOs were, in turn, expected to conduct orientation meetings with the health facility staff and distribute the documents to the health facilities. Health facility staff were expected to disseminate the information to the women during ANC health promotion classes. Both provincial and district managers explained that they used to conduct regular joint supervisory visits in the health facilities to ensure the documents on the new PNC schedule were disseminated to the health facilities and that health facility staff were oriented. Joint supervisory visits also served to confirm whether mothers were educated about the new guidelines during the health promotion classes which women attended when they came for ANC visits.

“*We received the new PNC guidelines from MoH and distributed them to the districts. We also trained the MCH coordinators from all the districts*” – Eastern Provincial MCH manager, IDI participant

“*We conduct monthly supervisory visits in the districts and health facilities to ensure that staff are oriented on the new guidelines”* – Southern Provincial MCH manager, IDI participant

### Evidence-based practices for routine PNC and management of complications

Although most participants perceived PNC services to be beneficial, they expressed limited knowledge about the actual services women and their babies were expected to receive at each visit. In addition, a stark difference in knowledge about PNC services was noted between different types of FGD participants. Women and SMAG members were generally more aware of the package of PNC services − including examination for postpartum complications such as bleeding and infections for the mother and her new-born; HIV and STI testing for the mother, and transmission prevention medication, if relevant, for the mother and neonate; breastfeeding and general hygiene; baby vaccinations, counselling on family planning and PNC-related health promotion messages. They also mentioned physical examination for the mother including blood pressure and pulse rate check-up; examination for fever, bleeding and foul vaginal discharge breast and nipple examination, anaemia and nutritional status. Other services for the baby were examination for signs of infection including fever, high pulse rate, state of the umbilical stamp, sucking patterns, excessive crying as well as general physical examination.

However, most men and elders were not clear on the actual package of PNC services that mothers and infants were expected to receive at each visit. For example, most fathers and community elders could only say that women and their infants need to go for examination and check-up, and that mothers were expected to receive health messages.

“*The woman is monitored for danger signs such as blood pressure, pulse rate, state of the uterus and cervix, cervical tears and bleeding. The baby is examined for skin colour, breathing, crying, sucking and other things. If both the mother and baby are ok, they are discharged and asked to return after 6 days*” - Mother, FGD participant, Intervention site, Kalomo district

*“Both the mother and baby are examined for complications. The mother is examined for fever, bleeding and foul vaginal discharge. The examination of the mother also includes blood pressure and pulse rate measurement, breast and nipple examination, anaemia and nutritional status. The baby is examined for signs of infection including fever, high pulse rate, state of the umbilical stamp, sucking patterns, excessive crying as well as general physical examination” -* District MCH manager, Nyimba District

While most community members and facility staff were equivocal on the quality of PNC services, provincial and district health staff, including a few mothers, generally expressed positive views on the quality of PNC services. Reasons for positive views were the short waiting time (i.e. less than 1 h) before they are attended to by healthcare staff, the availability and dissemination of the new PNC guidelines, trained health facility staff, availability of delivery rooms, and regular supply of commodities. Another reason was the availability of a PNC examination room which assured privacy as only one woman was allowed to enter at a time. Most mothers also described the health promotion classes to be beneficial as they provided women with information on HIV prevention for both the mother and baby, family planning, feeding and vaccination for their babies and general hygiene.

In contrast, most community members perceived the PNC services to be of low quality. Specifically, women and men were concerned about the absence or small size of PNC rooms and limited privacy and confidentiality in some health facilities. After delivery, women described being made to rest together in one small room, which in some instances only had one bed; other women had to rest in general wards or maternity waiting homes, which they felt put them and their infants at increased risk of infection. Although women were required to stay at the health facility after delivery before they could be discharged, women complained that, sometimes nurses were not available to help women if they developed complications like high blood pressure and/or bleeding after delivery. They also complained of long waiting time when they returned for PNC. Although vaccines were usually available, most mothers complained that they did not receive certain services like blood pressure check-ups, blood tests for anaemia and urine tests. They also explained that in some instances, mothers were not examined for postpartum complications such as bleeding, raised blood pressure and infection. Some mothers also explained that their neonates did not have their temperature or umbilical stumps checked.

*“Sometimes women can be alone in that room without anyone to help them if they develop complications like bleeding or fitting”* – Father, FGD participant, control site, Nyimba district

*“There is no room where we can rest with our babies; they make us rest in a small room which only has one bed. Sometimes there is more than one woman. So they take us to the other ward or mothers’ shelter”* - Mother, FGD respondent, intervention site, Nyimba district

### Respect, preservation of dignity and emotional support

Community members and health systems staff had mixed views on how mothers were treated during PNC visits. Most health managers, SMAG members, health staff and mothers expressed positive views on provider attitudes during PNC services. They explained that during health education classes, information was provided on the health of the mother and infant and that mothers were treated with respect during the PNC visits. In contrast, some mothers, fathers, and community members complained of poor treatment of women when they went for PNC visits with major reported forms of mistreatment including verbal abuse, disrespect, negative or condescending attitudes, failure to meet professional standards of care, and poor rapport between women and providers. Moreover, respondents explained that women who delivered at home found it difficult to go for PNC services because of the condescending attitude of health staff for having not delivered at the health facility. They explained that, in some circumstances, health staff disrespected them, sent them back home, or demanded payment before they attended to the mother and infant, or issued an under five clinic card or birth certificate. Some mothers and community members complained of long waiting times and the poor state or lack of PNC facilities which affected privacy and dignity of the women and provision of optimal PNC services.

“*Some nurses insult us when we come for PNC visits. They don’t respect us*” - Mother, FGD participant, Intervention site, Kalomo District

*“Some women go back before nurses see them because they have to find transport to go back” -* SMAG member, FGD participant, Choma District

### Functional referral system, motivated human and essential physical resources

Service providers and users were unanimous on the various health system challenges which affected provision and quality of PNC services. They explained that stock-outs of essential medicines, supplies and equipment, staffing constraints and non-availability of functional referral services for women with postpartum complications like bleeding, infection and pre-eclampsia affected the provision and quality of PNC services.

SMAG members and service providers explained that most health facilities had no functional ambulances and that in case of an emergency, health facility staff either called for an ambulance from the district hospital (often an hour or two drive away) or asked family members to find a vehicle in the community. Moreover, participants explained that the long distances to the district hospitals and poor state of roads put women and infants at risk. Respondents recommended the improvement of referral services for the management of these postpartum emergencies.

Nevertheless, respondents had mixed views regarding the use of maternity waiting homes during PNC visits. Mothers interviewed from the sites with MWHs which had been either upgraded or newly constructed had a positive attitude towards this service. They explained that MWHs were beneficial, especially for those who lived far from health facilities and those who needed to spend more time for observation at the health facility following postpartum complications. On the contrary, most mothers from health facilities with old no MWHs complained that MWHs were generally in deplorable states within the communities and put the infants at risk of infection. They recommended the need for construction of special PNC wards to make it easy for mothers and infants to use the PNC services. They also emphasized the need to improve the staffing levels for midwives, supply of medicines, vaccines, and other consumables.

***“****Some women and their babies just go back [home] without receiving anything because there are no medicines for emergency care.” -* SMAG member, FGD participant, Choma District

*“There is ambulance here. We call the district for help if we have an emergency.” -* SMAG member, FGD participant, Kalomo.

*“They should build more rooms [at the health facility] for mothers to stay after delivery. Mothers with small babies should not stay in that dirty maternity waiting home. They can get an infection* - Elder, FGD respondent, Pemba district

“*We don’t usually go to stay in the maternity waiting home because the baby can get an infection. There are many people who stay there* – Mother, FGD participant, Kalomo district

## Discussion

Service providers and users in rural Zambia perceive PNC services to be beneficial for the mother and the newborn baby. However, lack of awareness of the new PNC guidelines and schedules, dysfunctional referral services, and several health system-related factors affect respondents’ perception of the quality of PNC services. Below we address the major components of the WHO quality of care framework related to PNC services that users and/providers find lacking.

Timely and accurate communication of health information to service users is a key component of PNC quality. Through effective communication of health message people become aware of available services and learn about the potential and actual gains from using the services. Our findings suggest that community members and health systems staff appreciate the benefits of PNC services. During these visits, women and their newborn baby receive important information about child nutrition, breastfeeding and care, childhood vaccines and immunization schedule, growth monitoring and promotion, under five clinic and family planning. They also receive information about disease prevention such as HIV, tetanus, malaria, and others. Nevertheless, our findings show that some rural Zambian women were often dissatisfied with the way health workers communicated health messages to them regarding the timing of PNC visits according to the new schedule. Lack of awareness about the new PNC guidelines made most mothers not follow the PNC schedule; very few reportedly returned for the second and third visits according to the schedule. Rather, they reported continuing to follow the old guidelines and return after 6 days and 6 weeks postpartum. Thus, when information is not available, mothers are ignorant about the potential benefits, they fail to use the services at all or in the optimal ways. Lack of awareness and discontent among some SMAG members and health facility staff who had not seen the new guidelines or schedule further highlights the importance of effective communication. By contrast, district and provincial health managers who had either seen the guidelines or participated in the training expressed knowledge and satisfaction with the communication process. Previous studies and systematic reviews [41, 42] have highlighted the importance of effective communication on service users’ perceptions of care. Shakibazadeh and colleagues [43] found that effective communication is a key component of maternal health care quality, emphasizing the need for women to receive information about maternal health services to make informed decisions, avoid information asymmetry, and maximise perception of health benefits and utilisation of health services. In this context, health education classes during the antenatal period remain an important time for health workers to interact with pregnant women, provide information on the benefits of PNC services, address mothers’ concerns, and communicate important information, including PNC guidelines and schedules [44]. This finding highlights the importance of communication of health information to service users and suggests that interventions focusing on improving PNC quality should address communication barriers and provide correct and timely information to service users.

Negative or disrespectful attitudes of healthcare providers are a widespread phenomenon in LMICs, affecting users’ perceptions of quality and utilization of services, along the continuum of maternal and intrapartum health services. Bohren et al. [45] concluded that experiences of mistreatment during PNC visits have far-reaching consequences for women and communities outside of the direct woman–provider interaction and that experiences and perceptions of mistreatment, low expectations of the care provided at facilities, and poor reputations of facilities in the community erode many women’s trust in the health system and impact their decision to return there for subsequent PNC visits or future pregnancies [46]. Unsurprisingly, and in accordance with longstanding literature [46]**,** our findings suggest that women prefer PNC services where mothers are treated with respect. In addition, respondents described women preferring healthcare services where the healthcare providers communicated in a respectful, confidential, and caring manner. In contrast, poor rapport between women and providers during PNC visits – especially for those who delivered at home − was widely reported. Description of disrespectful care included verbal and/or physical abuse and power imbalances between women and healthcare providers.

Our findings suggest that PNC services provided in rural Zambian health facilities fall below optimal standards for evidence-based practices. For example, our findings show that in some instances women were not happy about the way nurses who were not available to help them if they developed postpartum complications like high blood pressure and/or bleeding. Major reasons for the low perception of PNC service quality were long waiting time, not receiving services like blood pressure check-ups, blood tests for anaemia, infections and urine tests, mothers not being examined for postpartum complications such as bleeding, raised blood pressure and infection. Neonates not having their temperature or umbilical stumps checked was a source of discontent among some mothers. Respondents reported that in some instances, women and their infants − especially those who delivered at home − did not receive the required services when they returned for PNC visits. These factors were perceived to endanger the life of the mother and her baby and made them perceive the services to be low quality. These findings are similar to those found in other LMICs [47, 48] and highlight the general gaps in health systems in LMICs, including low staff levels, long waiting time, small sized PNC rooms providing inadequate privacy, and stock-out of essential medicines, supplies and vaccines, which contribute to non-adherence to evidence-based practice. Long waiting times, in particular, affect user perception of service quality and user satisfaction of services [49].

A major concern is the reported lack of timely referral services for emergencies. In the study districts, ambulances were generally parked at the district hospital, requiring health facilities to call for one to come or have patients’ family members find a vehicle in the community to serve as a taxi for the referral. If a woman experienced an emergency at home, she would first need to travel the long distances to the health facility, be assessed, and then potentially wait for the arrival of an ambulance (or community vehicle) to bring her to a higher-level facility that can manage a postpartum complication, resulting in a multiple hour or even day(s)-long process for emergency treatment. Long waits for ambulances, long distances to the district hospitals, and the poor state of roads put mothers and infants at an increased risk of death if they developed complications. Our qualitative findings on the perceived low quality of PNC services are consistent with previous studies in the region [50, 51]. In their study on quality of basic maternal health care functions in health facilities of five African countries (Kenya, Namibia, Rwanda, Tanzania, and Uganda), Kruk and colleagues [51] found that PNC services provided in primary healthcare facilities were of low quality. These authors reported crucial deficiencies in staffing, essential infrastructure including electricity supply, basic emergency procedures, referral systems, routine and emergency care practices. Similar findings were reported by Nesbitt and colleagues [52] in Ghana. These authors found that most health facilities scored low on most signal functions including performance of routine care signal functions, skilled health professionals, and functioning at emergency obstetric and new-born care (EmONC) level. They concluded that low quality PNC services contribute substantially to maternal and neonatal deaths. These findings are important and suggest that interventions focusing on improving maternal and newborn health outcomes should ensure functional referral systems, adequate staffing for skilled healthcare staff, availability of essential medicines for routine and emergency care of the mother and neonate [53].

Although MWHs do not directly improve the quality of PNC services, our findings suggest that MWHs are a potential strategy for encouraging women to stay for the 48 h postpartum for postnatal checkup. These findings corroborate previous studies [54–56]. For example, in their study on the effects of MWHs on the health workforce and maternal health service delivery in rural Zambia, Kaiser et al. [56] reported that women waiting at MWHs allow staff to monitor a woman’s final stage of pregnancy and labour onset, detect complications earlier, and either more confidently manage those complications at the health centre or refer to higher level care.

### Limitations

This study has several limitations. First, the findings focus solely on the experience of the FGD and IDI participants from ten selected health facilities, two provincial health offices and four districts which operated within the context of SMGL. Project-related interventions or factors may have influenced the study participants’ perceptions. Second, like all qualitative studies, our findings are based on a purposively selected sample, and cannot be generalised to other settings with different geographic and demographic contexts. Further research is needed to measure the quality of PNC services, assess what services are routinely missed, and identify factors which affect user experience. Moreover, this study could not establish a causal link between PNC service quality and maternal and newborn health outcomes. In addition, the long-term effects of MWHs on PNC service quality are not known. Further research with a longitudinal quantitative design is required to establish the causal pathway.

Nevertheless, we believe that, based on the qualitative methods we used employing both FGDs and IDIs comprising respondents with widely varied characteristics and backgrounds sampled from various facilities in different regions of Zambia, our use of the international quality of care framework to guide exploration of the subject under investigation, and the rigorous data analysis approach, these findings accurately reflect the service users’ and providers’ perspectives on the quality of PNC services in the selected health facilities.

## Conclusion

These findings highlight users’ and providers’ perspectives on PNC service quality and provide evidence for investing in interventions to improve service quality in rural Zambia. They show the importance of effective and improved communication about the PNC services and their benefits as well as raising awareness on PNC timing of visits in improving quality of PNC services. The findings also highlight the importance of respectful care, preservation of dignity and emotional support provided to mothers during PNC visits. Interventions should focus on addressing contextual health system challenges including staffing levels, supply chain for essential medicines and commodities, adequate PNC rooms for privacy and confidentiality, shortening waiting time, and ensuring functional referral system. It would be beneficial for future studies to focus on: a) measuring PNC service quality and its determinants using quantitative methods, and b) confirming the causal link between PNC service quality and maternal and newborn health outcomes using longitudinal quantitative methods.

## Supplementary Information

**Additional file 1.** Informed consent form/ template for intending researchers.

## Data Availability

The datasets used and/or analysed during the current study are available from the corresponding author on reasonable request and with permission from the ERES ethics committee.
