# Supplementary material for: Postnatal care services in rural Zambia: a qualitative exploration of user, provider, and community perspectives on quality of care
Source: BMC Pregnancy Childbirth. 2023 Jan 18;23:39. doi: 10.1186/s12884-023-05350-w (PMC9847069; doi:10.1186/s12884-023-05350-w)
Supplement: Supplementary file 1 — Additional file 1. Informed consent form/ template for intending researchers. [file 12884_2023_5350_MOESM1_ESM.pdf]

POSTNATAL CARE SERVICES IN RURAL ZAMBIA: A QUALITATIVE EXPLORATION OF USER, PROVIDER, AND COMMUNITY PERSPECTIVES ON QUALITY OF CARE

INFORMED CONSENT FORM/ TEMPLATE FOR INTENDING RESEARCHERS

Background

Hello, my name is ___________________________ I am working with the Maternity Homes Access in Zambia (MAHMAZ) project. MAHMAZ is a quasi-experimental controlled before-and-after intervention design trial implemented by Boston University and Right to Care Zambia to evaluate the impact of newly constructed maternity waiting homes (MWHs) on reducing the distance barrier and increasing access to quality maternal health care services for rural women in Zambia. The project does not directly address postnatal care (PNC) services offered in the health facilities. It constructs new MWHs or upgrade existing ones; they also provide beds for postnatal women if they want to remain close to the health facility for PNC. Zambia has a high maternal mortality ratio (MMR) of 252 deaths per 100 000 live births and neonatal mortality rate of 23.5 deaths per 1000 live births. Most maternal deaths occur during childbirth (11-17%) and the first 48 hours postpartum (50-71%) and are due to complications of pregnancy, delivery and unsafe abortions−including haemorrhage, hypertensive disorders, sepsis, and obstructive labour. Nevertheless, most maternal deaths are preventable when women have access to quality and timely postnatal care (PNC).

Purpose: The aim of this study is to explore user, provider, and community perspectives on the quality of PNC services provided in rural districts of Southern and Eastern Provinces, Zambia. The information on service

quality provided here will help fill the knowledge gap on the quality of PNC services in rural Zambia and help guide Ministry of Health (MoH) policy on and provision of PNC services in the country.

Process: You will be one of approximately 170 respondents asked to participate in this study that is collecting information about the quality of PNC services. To ensure that we don't miss or forget anything during the discussion, your responses to the various questions will be recorded on this phone/tablet. We will not write your name, instead we ask you to sign on this form, but we will keep your name and signature private. Responses to the various questions will be summarised, analysed and a report written. When we write reports from your responses, we will not show what you said nor are we going to use your name or signature at any time.

Potential Benefits: You will receive no direct benefit from your participation in this discussion. However, your participation may help understand, from empirical evidence, the user, provider, and community perspectives on the quality of PNC services provided in rural districts of Southern and Eastern Provinces of Zambia. The information may help fill the knowledge gap on the quality of PNC services in rural Zambia and help guide Ministry of Health (MoH) policy on and provision of PNC services in the country.

Risks and Discomforts: The risks of taking part in this study are that other people will hear your responses. It is important that you do not share anything that you are not comfortable with. If you or someone in your family had a bad experience, it may be difficult or uncomfortable to remember or share it. You do not have to respond to any question unless you feel comfortable doing so. You are free to stop the discussion at any time if you need to.

.

Alternatives: You can choose not to take part in this discussion. If you decide not to take part or withdraw from this discussion, you will not suffer any penalty or lose any benefits to which you are entitled.

Participant Costs and Payments: You will not be paid to participate in this discussion. There are no costs to you for participating.

Participant's Rights: By agreeing to participate in this discussion, you do not waive any of your legal rights. Giving consent means that you have heard or read the information about this discussion and that you agree to participate. You will be given a copy of this form to keep. If you have questions or concerns at any time, you can contact the Principal Investigator,

Dr Sialubanje Cephas
Ministry of health
Chainama College of Health Sciences
P.O. Box 33991
Lusaka
Mobile: 260-977-441273,
email: csialubanje@yahoo.com,

or 95 or any of the staff from the ERES Converge Ethics Committee on +260-21-1-230581

Right to Refuse or Withdraw: Taking part in this discussion is voluntary. You have the right to refuse to take part. If you decide to be in this discussion, and then change your mind, you can withdraw from it at any time and to skip questions you may deem personal or otherwise without any repercussions. Your participation is voluntary. If you choose to take part, you have the right to stop the discussion at any time.

Confidentiality: The results of this study will be kept strictly confidential and used only for research purposes. Your identity will be concealed in as far as the law allows. Your name will not appear anywhere on the coded forms with the information. Paper and computer records will be kept under lock and key and with password protection respectively. The interviewer has discussed this information with me and offered to answer my questions. For any further questions, I may contact the Chairperson, ERES Converge on the following details___________________

STATEMENT OF CONSENT/ASSENT

.... has described to me what is going to be done, the risks, the benefits involved and my rights regarding this study. I understand that my decision to participate in this study will not alter my usual medical care. In the use of this information, my identity will be concealed. I am aware that I may withdraw at anytime. I understand that by signing this form, I do not waive any of my legal rights but merely indicate that I have been informed about the research study in which I am voluntarily agreeing to participate. A copy of this form will be provided to me.

Name:…………………………Signature of participant ……………………..Age..……………
Date (DD/MM/YY)……………………………

Name of Witness……………………………….. Signature of Witness………………………..
Date (DD/MM/YY)……………………………………………

Name…………………………Signature of parent or guardian for minors ……………………
Date(DD/MM/YY)…………………………

Name…………………………………..Signature of Interviewer ………………………….Date (DD/MM/YY)………………………...

If you have any further questions please contact the ERES Converge Ethics Committee

Telephone: 230581

By signing below you are agreeing to participate in the discussion which indicates that you have read this consent form (or have had it read to you), that your questions have been answered to your satisfaction, and that you voluntarily agree to participate in this research study. You may keep a copy of this for your records.

Signature or thumb print: ____

Date: ____

Signature of Impartial Witness: ____

Date: ____
